# Characterization of toxin-antitoxin systems from public sequencing data: A case study in *Pseudomonas aeruginosa*

**DOI:** 10.3389/fmicb.2022.951774

**Published:** 2022-08-16

**Authors:** Zehan Dai, Tianzhi Wu, Shuangbin Xu, Lang Zhou, Wenli Tang, Erqian Hu, Li Zhan, Meijun Chen, Guangchuang Yu

**Affiliations:** Department of Bioinformatics, School of Basic Medical Sciences, Southern Medical University, Guangzhou, China

**Keywords:** genome mining, *Pseudomonas aeruginosa*, toxin-antitoxin system, pangenome, case study

## Abstract

The toxin-antitoxin (TA) system is a widely distributed group of genetic modules that play important roles in the life of prokaryotes, with mobile genetic elements (MGEs) contributing to the dissemination of antibiotic resistance gene (ARG). The diversity and richness of TA systems in *Pseudomonas aeruginosa*, as one of the bacterial species with ARGs, have not yet been completely demonstrated. In this study, we explored the TA systems from the public genomic sequencing data and genome sequences. A small scale of genomic sequencing data in 281 isolates was selected from the NCBI SRA database, reassembling the genomes of these isolates led to the findings of abundant TA homologs. Furthermore, remapping these identified TA modules on 5,437 genome/draft genomes uncovers a great diversity of TA modules in *P. aeruginosa*. Moreover, manual inspection revealed several TA systems that were not yet reported in *P. aeruginosa* including the *hok-sok*, *cptA-cptB*, *cbeA-cbtA*, *tomB-hha*, and *ryeA-sdsR*. Additional annotation revealed that a large number of MGEs were closely distributed with TA. Also, 16% of ARGs are located relatively close to TA. Our work confirmed a wealth of TA genes in the unexplored *P. aeruginosa* pan-genomes, expanded the knowledge on *P. aeruginosa*, and provided methodological tips on large-scale data mining for future studies. The co-occurrence of MGE, ARG, and TA may indicate a potential interaction in their dissemination.

## Introduction

Toxin-antitoxin (TA) system is a group of genetic modules widely occurring in prokaryotes. A typical TA system consists of a toxin component, providing toxicity to kill the host cells, and an antitoxin, conferring immunity to the toxin protein/RNA (Unterholzner et al., 2013). They were first reported on plasmids (Gerdes et al., 1986), and later abundant TA systems were found to be on chromosomes (Tsilibaris et al., 2007; Saavedra De Bast et al., 2008). The TA system is a multi-role player providing several biological functions in bacterial life. The first TA system was reported to be inducing post-segregation killing (PSK) to ensure stable plasmid maintenance in the host population (Gerdes et al., 1986). Similar to those found on the plasmid, the chromosomal TA systems have proven to stabilize the mobile elements, integrative conjugative elements (ICEs), pathogenicity islands, and super-integrons (Pandey and Gerdes, 2005; Christensen-Dalsgaard and Gerdes, 2006; Mazel, 2006; Qiu et al., 2006; Szekeres et al., 2007; Tsilibaris et al., 2007; Wozniak and Waldor, 2009). Besides, the TA systems were reported to be involved in virulence enhancement (Ramage et al., 2009; Norton and Mulvey, 2012; De La Cruz et al., 2013), biofilm formation (González Barrios et al., 2006; Kim et al., 2009), and induction of bacteriostatic persistence state (Page and Peti, 2016). Also, the TA systems have been drawing attention to their role in antimicrobial resistance (Page and Peti, 2016; Vogwill et al., 2016; Yang and Walsh, 2017).

Due to the bacterial inhibitory activity of toxin components, the TA systems have been proposed as an alternative antibacterial strategy to traditional antibiotics. Though the antibacterial activity of the TA was activated under limited conditions, the lethality of most of the toxins is confirmed in the absence of corresponding antitoxins (Harms et al., 2018). Besides, some features make the toxins a great option as a new type of antibacterial method. The toxins are encoded by single genes, which are relatively short (80–630 bp), instead of large gene clusters (Sevin and Barloy-Hubler, 2007). The simple genetic structures make them easy to predict and manipulate, which is ideal for genetic engineering. It is also reported that some TA systems were limited to specific types of bacteria (Fozo et al., 2010), combined with the fact that most of the known toxins were mostly cytosolic, except for some type III and type VII toxins (Song and Wood, 2018), these TA systems may be of the narrow antibacterial spectrum (Williams and Hergenrother, 2012; Równicki et al., 2020). Several strategies have been raised for artificially activating the toxicity of the TA system including interfering with TA complex formation, accelerating the antitoxin protein degradation by activating Lon protease, repressing transcription or translation of the antitoxin, and delivering recombinant toxin RNA/DNA (Williams and Hergenrother, 2012; Lee and Lee, 2016). Successful cases employing some of these strategies have shown the potential of applying the TA as antibacterial weapons (Christensen et al., 2004; Lioy et al., 2010; Równicki et al., 2018; López-Igual et al., 2019).

Based on the regulatory mechanism, the TA systems could be classified into eight classes. The type I–VI TA systems have been well documented as six main classes of TA systems (Wang et al., 2020). The type I and type III TA systems are modules consisting of RNA antitoxins and protein toxins while type II, IV, V, VI, and VII, were protein-protein TA modules (Singh et al., 2021). Type I antitoxins are antisense RNA that binds the toxin mRNA to inhibit toxin gene from translation while the type III antitoxin RNA directly interacts with toxin protein, forming a macromolecular complex to eliminate the activity of toxins (Fineran et al., 2009; Blower et al., 2012). The type II modules were the most documented system in the big family of TA systems; their antitoxins counteract the toxins by forming a protein-protein complex to inhibit the toxin lethality (Goeders and Van Melderen, 2014). Unlike type II TA systems, the type IV, V, and VI antitoxin protein suppress the toxin by competitive binding substrate instead of interacting directly (Masuda et al., 2012), functioning as enzymes degrading toxin mRNAs (Wang et al., 2012), and mediating proteolytic degradation (Aakre et al., 2013; Markovski and Wickner, 2013). The type VII TA system is a newly described system proposed in 2020 (Wang et al., 2020) whose antitoxins function as enzymes to neutralize the toxin activity. The VIII system is the only TA family in which the toxins are RNA and the antisense antitoxin sRNAs neutralize the toxins by directly binding them (Choi et al., 2018). The first sample of VIII was reported in 2018 (Choi et al., 2018) but was formally proposed as VIII TA in 2020 (Song and Wood, 2020).

The most widely studied class of all TA modules is the type II system. According to statistics of TADB2, a TA database that curated sequences and annotation through an exhaustive survey on 586 papers, the total numbers of TA loci (experimentally validated) collected were 47, 105, 7, 1, 1, and 1 of type I, type II, type III, type IV, type V, and type VI, respectively (Xie et al., 2018). The type II TA loci account for most of all the available TA references, let alone 6,088 records generated by *in silico* prediction, which accounts for over 95% of the whole TADB2. It is hard to conclude that type II is the most abundant class in nature since the bias is caused by available reference sequences and the limited species of which TA systems have been systematically characterized. The diversity, richness, and preference of TA genes depend on complicated factors. A distinct distribution pattern of TA was observed among the Actinobacteria, Bacteroidetes, Firmicutes, and Proteobacteria (Akarsu et al., 2019), which indicates a distribution preference among different taxa. The type II TA modules were reported to be more abundant in pathogenic bacteria than non-pathogenic ones except for the obligate intracellular bacteria (Pandey and Gerdes, 2005; Lobato-Máarquez et al., 2016; Kang et al., 2018). The type III TA modules were reported to be in high abundance in intestinal microbiomes (Kang et al., 2018). Though there are only a few loci reported in the type VIII system, it is presumed to be widespread throughout the family, Enterobacteriaceae (Singh et al., 2021).

*Pseudomonas aeruginosa* is a pathogenic bacteria widely distributed in nature. Being an opportunistic pathogen, it could cause a variety of acute infections (Maurice et al., 2018). The intrinsic resistance of *P. aeruginosa*, combined with its adapting ability make this species easily develop resistance against drugs (Munita and Arias, 2016; Hall et al., 2018; Azam and Khan, 2019). Especially, it was pointed out that the carbapenem-resistant *P. aeruginosa* strains need extra attention for their rapid dissemination mediated by mobile genetic elements (MGEs) (Yoon and Jeong, 2021). The TA systems in *P. aeruginosa* have been reported to play key roles in plasmid maintenance (Bonnin et al., 2013), biofilm formation (Williams et al., 2011; Wood and Wood, 2016), drug resistance (Bonnin et al., 2013), virulence (Garcia-Contreras et al., 2008; Ren et al., 2012; Wood and Wood, 2016), and long-term infections (Mulcahy et al., 2010). The diversity and abundance of TA modules in *P. aeruginosa* have been explored in clinical isolates (Fernández-García et al., 2016; Andersen et al., 2017). The TA modules reported in *P. aeruginosa* included ParAB, TOX1/TOX2, T/AT1-2, RelBE, HigBA, GraTA, MazEF, VapBC, YefM/YoeB, Hha/TomB, and PasTI (Fernández-García et al., 2016). Another study characterized TA modules at the domain level and uncovered 26 combinations of toxin–antitoxin pairs (Andersen et al., 2017). All TA classes in both studies described are of type II TA modules, while TA modules of other classes in *P. aeruginosa* were not well documented; re-investigation of TA modules of all classes in *P. aeruginosa* may lead to a better understanding of the TA family in the species, *P. aeruginosa*, especially given the circumstance of abundant reference genomes and sequencing data in public databases. A small survey conducted by our group revealed that 5,095 complete or draft genomes of *P. aeruginosa* were recorded and 15,357 genomic HTS data were openly accessible in the NCBI Sequence Read Archive (SRA) database (Leinonen et al., 2011). These resources provide a wealth of property for data mining and reanalyzing HTS data might lead to new findings.

In this study, we selected *P. aeruginosa* as the research object, aiming to search for TA homologs from the unexplored parts of *P. aeruginosa* pan-genome through *in silico* analysis. Genomic sequencing data of the selected isolates from *P. aeruginosa* were downloaded from the NCBI SRA database (Leinonen et al., 2011), reassembled, and gone through a homology-based search in reference to the TADB database (Xie et al., 2018). Orphan genes or TA systems not documented in *P. aeruginosa* were gone through rule-based evaluation. The identified TA modules, combined with the original TADB references were mapped back to the complete genomes or draft genomes of 5,437 strains, collected from the NCBI genome database to uncover the TA systems in the *P. aeruginosa* population. Furthermore, we also annotated the MGE and antibiotic resistance gene (ARG) on the TA-carrying sequences. The diversity, abundance, and prevalence of TA families will be characterized, and new findings of TA modules on *P. aeruginosa* will be discussed. Some methods were raised in this study to automate TA characterization, and the difficulties and unresolved problems in the analysis will be discussed.

## Materials and methods

### Overview of the analysis workflow

**FIGURE 1:**
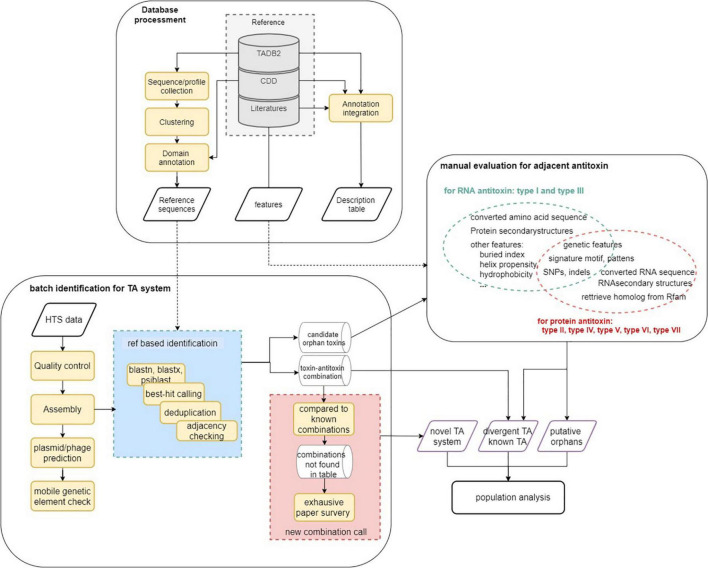
Schematic overview of the analysis workflow. Step 1. Database curation and setting-up for downstream analysis. Step 2. TA identification using homology-based strategy. Step 3. Rule-based TA identification (for orphan) and functional inspection. Step 4. Update the reference data using identified TA systems and perform homologous TA module characterization in *P. aeruginosa* population.

The analysis workflow of this study is described in Figure 1. In general, reference databases were curated for the following analysis modules followed by homology analysis and manual evaluation (on putative orphan genes and TA modules not yet reported in *P. aeruginosa*). Finally, the identified TA modules were used to update the reference data set and to identify TA systems on the 5,437 genomes to characterize TA distribution in the population. Key parameters of the software used and a brief explanation are listed in Supplementary Table 3.

### Data collection and genome assembly

To select qualified genomic sequencing data of P. aeruginosa from the NCBI SRA database (Leinonen et al., 2011), data table containing meta data of all Biosamples/Bioprojects was acquired and checked. We manually inspected all the available information and selected isolates following the principles: (1) must be genomic data; (2) avoid model strains (the PAO1) and samples of the same strains; (3) avoid samples from artificial mutation; (4) select Bioproject aiming at exploring strains from different sources (isolates from different geographical location, niche, and host); (5) must be isolates and single clones. A summary of the selected data is listed in Supplementary Table 2. Sequencing data in SRA format were downloaded and converted to FASTQ format with the assistance of the NCBI SRA toolkit (Leinonen et al., 2011) or the Aspera Connect v 4.0.2.^1^

Regarding assemblies for later-on population analysis, a total of 5,432 genome sequences of *P. aeruginosa* were downloaded from the NCBI Genome database, including assemblies of complete circular replicons and draft genomes (scaffold and contigs). Among all the strains, 52 included complete plasmid sequences and they are also included in this study. For assemblies at scaffold/contig level with no corresponding plasmid information, all sequences were gone through plasmid/phage prediction with viralVerify (Antipov et al., 2020) as will be further described in Section “Plasmid and bacteriophage sequence prediction and homology search.” Information of all the selected reference genomes is summarized in Supplementary Table 1.

To conduct quality control on the sequencing data, the Fastp (Chen et al., 2018) was employed. The base correction was triggered for pair-ended data, low-quality tail, and adapters were trimmed (see Supplementary Table 3 for all parameters). The clean reads were then assembled into contigs using Spades (Prjibelski et al., 2020).

### Plasmid and bacteriophage sequence prediction and homology search

The plasmid and phage contigs were predicted by viralVerify v0.1.1 (Antipov et al., 2020) by which all contigs were classified into different subsets: chromosome, putative plasmid, and putative bacteriophage (including the free form and integrative form). Before conducting the prediction, we preliminarily evaluated the performance of viralVerify with test sets by checking the true positive rate (TPR) of all predictions. The test sets included the virus subset (Vref_db) and the plasmid subset (Pref_db) of the NCBI RefSeq database (GenBank release version 223), and 296 chromosomal genomes of *P. aeruginosa* (Supplementary Table 2) of complete replicon (PA_ref_db). The result showed that the TPR was 91, 85.1, and 100% for predicting Pref_db, Vref_db, and PA_ref_db, respectively. Based on this, we propose the viralVerify as a qualified tool for classification.

The novelty of all contigs was confirmed by aligning sequence against reference database using blastn embedded in the BLAST+ (Camacho et al., 2009). For chromosomal contigs, the contigs were aligned against PA_ref_db; for plasmid and the uncertain plasmids sequences; aligned against Pref_db; for putative bacteriophage sequences, and were aligned against Vref_db.

To automate the best-hit calling for raw blast results on a large scale, we introduced a statistic named “adjusted identity of merge hits” (AIMH). Given a query contig Q and a subject sequence S (reference matched by query), equal to or more than one hit (H) was observed, and the AIMH was calculated as below:


$$\mathrm{AIMH}=\frac{\sum_{i=1}^{n}I_{i}}{n}*L$$

Where I is the sequence identity of an alignment.

The L indicates the maximal value of non-redundant merged coverage between the query and the subject. The value of L can be calculated as follows:


L=max(cov(Q),cov(S))

The cov(Q) is calculated by dividing the sum of the accumulated alignment region (deduplicated regions without overlap) size on the query by the total query size. The cov(S) is calculated in the same way.

The AIMH is designed for calling the best hit in batch data without further human interpretation and overcoming the difficulties of comparing local hits with only bit-score and *E*-value.

### Identification of toxin-antitoxin systems

The TA database TADB2 (Xie et al., 2018) was used as a reference for identifying candidate TA systems which includes TA systems from type I to type VI. References of type VII (Wang et al., 2020) and type VIII (Singh et al., 2021), as well as several TA systems, were manually curated. In TADB2, only a small part of the reference was experimentally validated while most of the records are generated by *in silico* prediction. A summary of reference loci with experimental proof was recorded in Supplementary Table 7. The BLAST+ and Diamond v0.9.30.131 (Buchfink et al., 2015) were used for the similarity search.

Extra information was collected for further evaluation of some putative TA systems. The multiple sequence alignment (MSA) was conducted using MUSCLE (Edgar, 2004) or the Smith-Waterman algorithm (Smith and Waterman, 1981) embedded in the comprehensive workbench tool, UGENE 34.0 (Okonechnikov et al., 2012). For divergent or orphan toxins, flanking sequences were extracted, and the domain was predicted using the Conserved Domain Search (Lu et al., 2020). The vsfold5 (Dawson et al., 2007) was employed to predict secondary structure and pseudoknot. The RaptorX (Peng and Xu, 2011) and SWISS-Model^2^ were used to predict linear helix structures and protein 3D models. These results were manually inspected, serving as additional hints for evaluating the TA result. For orphan toxins, this information serves as additional features to further confirm possible novel antitoxins. Redundant information (inner-contig and inter-sample duplication) was filtered using customize Shell/Python scripts.

The final result was manually checked, and results with ambiguous annotations were further evaluated by the online blastp function implemented on NCBI^3^ against the NR database, and the standalone rps-blast was implemented in BLAST+ against the CDD database (Lu et al., 2020). Additional annotations were added and the final results are summarized in Supplementary Table 3.

### Relativeness evaluation of the reference species

A statistic namely pairwise kinship distance (pKD) was introduced to evaluate the relative distance of two species based on a taxonomy tree. The pKD is calculated by comparing lineages of two species. A qualified lineage for calculating pKD should consist of seven levels: kingdom (L1), phylum (L2), class (L3), order (L4), family (L5), genus (L6), and species (L7). Given a query species A and reference species B, the pKD was calculated as below:


$$\mathrm{pKD}\left(A,B\right)=\frac{2\times r\left(\mathrm{LCA}\right)-r\left(A\right)-r\left(B\right)}{12}\times 100\%$$

Each rank of a lineage was assigned a numeric label, *r* from −1 to −7. The edges between the L7 node of A (or B) and the lowest common ancestor (LCA) were counted, and the raw distance from each species to LCA was measured by subtracting *r*(A) or *r*(B) from *r*(LCA) followed by summing up both the values. The sum was then divided by the maximum distance two species could reach, which is 12, to map the raw distance in the range of [0, 1]. All lineage information of reference species was extracted from the NCBI taxonomy database (NTD) (Schoch et al., 2020). The python package, Taxonomy v0.7.1^4^ was employed to parse the dump files from NTD and return the lineage information.

To reduce the bias introduced by poor lineage annotations in NTD, a correction method was developed to pre-process the lineage information before pKD calculation. Unqualified lineages contain missing ranks and additional ranks which do not belong to any of the seven levels of a qualified lineage as defined above (e.g., subgroups between levels); such annotations would lead to inaccuracy. Regarding the lineages with missing ranks, the lineages will be dropped or corrected based on different scenarios: (1) if only one missing node is present and it is at L7, this lineage will be considered as a complete lineage; (2) else, check the nodes between the highest missing node (HMN) and the observed LCA (oLCA); if the rank of HMN is higher than oLCA, this lineage will be seen as a complete lineage; (3) if the HMN rank is lower than oLCA, and a node exists in between oLCA and HMN, the lineage will seem like a complete lineage; (4) if the HMN rank is lower than oLCA and no nodes exist in between oLCA and HMN, this lineage will be dropped since it cannot exclude the possibility that the real LCA is the same as HMN instead of oLCA.

### Contamination exclusion and integration element determination

To ensure all the data are true of *P. aeruginosa*, or to confirm possible contamination from other species, the metagenome composition was checked. The MetaPhlAn3 (Beghini et al., 2021) was employed to collect taxa information in the HTS data or draft genome. The reference database was the default database curated in MetaPhlAn3 and all parameters were set as default except adding the “add-virus” tag. Given taxa assignment of a sample, if the relative abundance was over 1%, this species is considered a valid species, and if the bacterial species other than *P. aeruginosa* were identified, this sample was considered to be a contaminated one.

The sequences on which TA was identified were aligned against all Pref_db, Vref_db, and PA_ref_db to confirm whether they are integrative elements in chromosome with blastn. Those matching both chromosomes and plasmid/phages reference genomes or predicted as plasmid/phages by viralVerify were considered integrative elements.

The circularity of putative plasmid or phage sequences was evaluated with reference to a previous study (Jørgensen et al., 2014). In brief, the beginning and end of a circular sequence generated by *de novo* assembly should overlap with each other; thus it can serve as a hint to determine circular sequences or free forms of integrative sequence. The predicted plasmid or phage sequences (generated by viralVerify) were aligned to each other to see if they overlap; matches of at least 50 bp with over 40% identity were observed, and these sequences were considered to be circular.

The copy number was also used as hints for plasmid and phage identification. In extreme cases, circular plasmids and phages have much higher copies than chromosomes. The average nucleotide coverage (ANC) of these sequences was calculated with the assistance of hisat2 (Kim et al., 2019) to determine whether a free form of integrative plasmid/prophage exists. The ANC of five housekeeping genes, the 16S rRNA gene, *rpoB*, *gapA*, *tufA*, and *gryB* was calculated to represent the average copy level of all sequences in each SRA data. If the ANC of the sequences with TA was twice higher as the highest ANC among the five genes, these sequences were considered as high coverage.

Combined with all the information mentioned, contamination was manually determined. Sequences with short size (<500), low coverage, and non-integrative, were considered as possible contamination and removed from this study. This information is added to Supplementary Table 4.

### Map identified toxin-antitoxin modules to model strains

The genome sequence of *P. aeruginosa* PAO1 (assembly accession number GCF_000006765.1) and *P. aeruginosa* PA14 (GCF_000014625.1) were downloaded from the NCBI Genome database. The final identified TA modules were aligned against the chromosomal genome sequence of the model strain, *P. aeruginosa* PAO1 and the *P. aeruginosa* PA14 to find out homologous regions in these strains. Detailed information on the identified homologs in *both* strains is summarized in Supplementary Table 6.

### Mobile genetic element and antibiotic resistance gene identification

The MGE homologs were identified with reference to the ICEberg database (Liu et al., 2019). This database consists of 50 ICEs 31 integrative and mobilizable elements (IMEs), 522 actinomycete ICEs AICEs (AICEs), 111 ***cis***-mobilizable elements (CIMEs), and 662 type IV secretion system (T4SS) type ICEs, respectively. Several steps for automating valid MGE region calling based on blast alignments: (1) hits with less than 5% sequence coverage over reference were filtered; (2) for global alignment (coverage on reference sequence ≥ 90%), the longest high scoring pair (HSP) with the highest identity to was chosen; (3) for local alignment (multiple shorter HSPs refer to the same reference MGE), the overlap or containment hits were merged first, then all the non-overlap HSPs had gone through distance check; that is, using a dynamic cutoff (20% of the reference size) to a pair of HSPs were distant or adjacent, to determine distant HSP groups. Finally, given an HSP group, if the accumulated size of all non-overlap HSP members were less than 120% of the reference size, the HSP group was considered an MGE region.

For ARG detection, nucleotide sequences of ARG genes from the CARD database (McArthur et al., 2013) were collected and served as references. The ARG calling was conducted the same as the MGE detection as described above.

## Results

### Overview of the toxin-antitoxin modules uncovered in the HTS data

**FIGURE 2:**
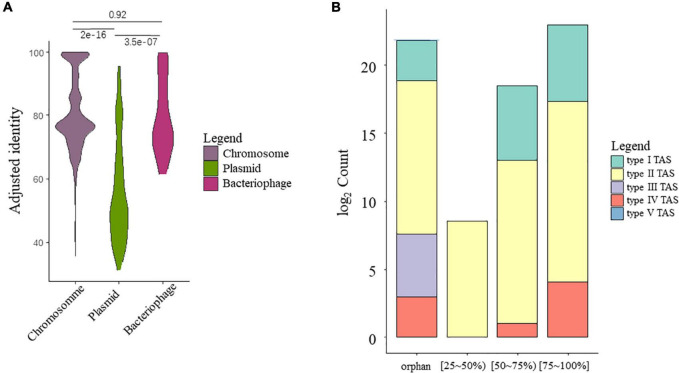
Overview of the putative TA gene hits. **(A)** Identity preference of putative TA gene hits toward different categories. Pairwise significance tests were conducted using Kruskal–Wallis test. **(B)** Counts of TA gene hits grouped by mean AIMH scores and TA classes.

A total of 16,963 TA gene hits (including sole toxin homologs) were identified from HTS data of all strains, among which 94% were identified on chromosome sequences, while small fractions of TA systems were identified in the putative bacteriophage (26, 0.15%) or plasmids (935, 5.51%). Interestingly, the TA hits found on chromosomes and putative bacteriophages showed higher homology to the reference compared to those on plasmids, (Figure 2A), as supported by the Kruskal–Wallis test (χ^2^ = 84.535, *P* = 2.2e-16). The identity score range between TA-chromosome and TA-bacteriophage was roughly the same. As active MGEs, the bacteriophage and plasmid should have higher mutation rates, let alone the genes on them. This is further confirmed by checking the distribution of TA count by identity grouping, as most of the TA from the putative plasmid subset belongs to a low similarity group (AIMH lower than 50%) (Supplementary Figure 1).

A summary barplot was created to visualize the overall TA system counts grouped by TA classification and identity (Figure 2B). Regarding the TA class, most of the TA gene hits belong to type II TA systems, with 16,808 in total, followed by type I (102), type IV (27), type III (24), and type V (2). Most of the TA gene hits have similarities of over 50%, where only a small fraction has low homology. A large number of orphan toxin homologs were also observed. The orphan toxin shows a similar pattern as the complete TA systems, as with most of the orphans belonging to the type II TA systems, followed by type I and IV TA systems. Interestingly, all type III homologs were orphan hits with no adjacent antitoxin.

In general, the abundance of type II TA homologs is expected since it is the most abundant TA system uncovered from nature so far. This may be a bias introduced from the unbalanced reference database. In view of the TA families, most type II gene hits uncovered at this stage were of reported TA systems, as documented in two studies (Fernández-García et al., 2016; Andersen et al., 2017). A description of all the TA families reported is uncovered in Supplementary Table 7. A large number of divergent TA systems and orphan toxins were uncovered beside the homologs highly similar to the known TA systems. The divergent and the possible new TA systems, as well as the orphan toxins, will be further discussed later.

### Toxin-antitoxin systems not yet reported in *Pseudomonas aeruginosa*

#### hok-sok-mok

Two orphan *hok* homologs of type I, the dtas1_hok and the dtas2_*hok*, were identified from two distinct chromosomal contigs of the sample SRR10338934. The dtas2 was omitted for further analysis of the low homology (34%) toward reference and missing a convincing toxin domain. The dtas1_hok nucleotide sequence was 73% identical to the reference *hok* T6324, combined with the result of domain scan, open reading frame (ORF) prediction, and MSA, we manually confirmed the complete *hok* homolog with the similar size of T6324 (155/161 bp) (Supplementary Figure 2A). A *mok*-like region containing almost the whole *hok* region was also confirmed by ORF prediction (Supplementary Figure 2B).

An attempt to find the “missing” antitoxin led to the finding of a *sok*-like antitoxin. The type I TA system is featured by antisense RNA antitoxin which binds the toxin mRNA transcript to disrupt the toxin translation (Gerdes et al., 1986; Fozo et al., 2008). The Shine-Dalgarno (SD) sequences on the TIRs of *mok* and *hok* were required for the full-size *hok* mRNA to form proper hyper-structure involved with expression regulation (Pedersen and Gerdes, 1999). Scanning the 1,000-bp flanks of dtas1_hok yielded an SD-like pattern “GGAG” which was identified by manual search on the TIR sequence of dtas1_*hok* (Supplementary Table 5; Supplementary Figure 2B). A short region identical to the *sok* target (sokT) of *flm* locus, a divergent form of *hok*, was also observed (Pedersen and Gerdes, 1999). This indicates a *sok* homolog since the *sok* locus was reported to overlap with *mok* locus (Gerdes et al., 1990). A homolog of *sok* was determined on the flank sequence of dtas1_*hok* containing these signatures through the database searching function of Rfam (Kalvari et al., 2021). The closest reference loci matching the dtas1_sok was the *sok* of Enterobacter sp. WP_7_1 (URS0000E30588_1907563) with an identity of 78%.

#### ralD-ldrD

Another type I TA module, assigned as dtas4_*ralD-ldrD*, was identified in SRR10338934. The 105-nt homolog dtas4_*ralD* and 66-nt dtas4_*ldrD* shared 85.07 and 62% global identity with the nearest reference TADB| T6332 and TADB| AT6339 (a homolog of TADB| AT6332). The genetic organization of dtas4_*ralD-ldrD* loci was similar to the *ralD-ldrD* reported in *Escherichia coli* K-12 (Kawano et al., 2002; Supplementary Figure 3A) though the beginning of *ldlD* mRNA cannot be precisely confirmed. Multiple putative promoters were recovered on the −60 to −160 upstream of the *rdlD* gene (Supplementary Figure 3A). Further MSA alignment against all 48 references in TADB2 reviewed that these sequences could be grouped into four unique sequence types based on homology (Supplementary Figure 3B) and dtas4_LdrD carries a distinct sequence pattern but preserves most of the conserved residues (Kawano et al., 2002). The only conserve amino acid difference was the aspartate on position 12 and the arginine on position 30. It is reported that the impact of conservation residues on LdrD has not yet been demonstrated (Nonin-Lecomte et al., 2021). The MSA of amino acids highlighting hydrophobicity reviewed that the sequential hydrophobicity pattern of dtas4_LdrD from position 13 to position 32 is similar to TADB| T6332 (Supplementary Figure 3C), which was experimentally confirmed to form α-helix structure (Kawano et al., 2002). The predicted protein secondary structure uncovered that the region 13–32 forms a reliable α-helix. The possible effect of the variance on dtas4_ldrD and its function will be discussed later.

#### cptA-cptB

A homolog of *cptA-cptB*, designated as dtas351, was uncovered from SRR10338934. The TA of dtas351 was 72/84% identical to the reference *cptA-cptB* genomic sequence (T6368-AT6368) from *E. coli* str. K-12 substr. MG1655. It is reported that the toxin *cptA* is featured by a transmembrane domain which may spatially limit the *cptA* activity only near the membrane (Masuda et al., 2012). The MSA coloring in the hydrophobicity scheme revealed that the amino acid replacement caused by single nucleotide polymorphism (SNP) did not reverse the hydrophobicity on the replaced sites (Supplementary Figure 3C). Secondary structure and 3D models predicted over the whole toxin sequences revealed that the α-helix structure was not altered in dtas351_CptA (Supplementary Figure 3D). Another homolog, assigned as dtas_352, was identified in a 317-nt contig from SRR11276951, the toxin CptA has similar predicted structure to dtas351 (Supplementary Figure 3E). But this sample were confirmed to be meta-genome, thus it’s unclear that whether it’s from *P. aeruginosa*.

#### cbeA-cbtA

Two false orphan YeeV homologs belonging to type IV were identified in the SRR10338934 and SRR11288271 assigned as dtas353, and dtas355, respectively. The dtas355-YeeV locate at the 5′ end of a contig; thus it is unsure whether it is a true orphan, while the sequence carrying dtas353 was a large chromosomal contig with a size of 416,000 bp. The dtas354_YeeV was highly similar to the reference, TADB| T1042. Though the online blastx against the NR database did not provide solid hints of any adjacent putative antitoxin, the followed CDD scan confirmed the presence of *cbeA* right next to the dtas353. The presence of *cbeA* indicated that the dtas353 were complete TA systems and reassigned as *cbeA-cbtA*.

#### ryeA-sdsR

Three *ryeA-sdsR* belonging to type VIII TA systems were detected from three published genomes. The contig CVWA01000245.1 carrying one of the homologs is a short sequence with only 213 bp, on which the TA module is incomplete; thus it is excluded for further discussion.

Similar to the known ryeA-sdsR, all sdsR homologs locate within and on the opposite strand of ryeA homologs (Supplementary Figure 6A). The border of the *ryeA-sdsR* homologs on GCA_001067615 and GCA_900683395 cannot be determined since no adjacent promoters and polyA were found. The nearest start codon locates 438 bp on the upstream of *ryeA-sdsR* regions, which is more likely from another gene locus. The whole regions of all *ryeA* sequences showed relatively low homology to each other (Supplementary Figures 6B,C) except the *sdsR* regions (Supplementary Figure 6C, from 140 to 246). It has been proved that a short region is required for the toxicity of SdsR; as it is highlighted in Supplementary Figure 5B, the 5′ staring 50 nt and the 3′ terminator sequences are dispensable and the deletion of these regions does not alter the toxicity of SdsR (Choi et al., 2018). Sequences on the toxicity-required region were identical, which is even more conserved than the dispensable regions. Combined with this information, it could be assumed that the product of this *sdsR* could be still functional, but whether the *ryeA* homologs found in this study can be transcribed or not remain questionable since the operon seemed to be truncated.

### Homologs of previously reported toxin-antitoxin systems

Abundant type II TA systems with high similarity to known reference TA systems were identified. No type V and type VI TA systems were identified. Most of the type II TA systems found in this study were recorded in previous studies. A total of 33 types of type II TA systems have been confirmed in *P. aeruginosa* (Fernández-García et al., 2016; Andersen et al., 2017; Chaudhary et al., 2018). Homologs of well-characterized TA systems include *parD-parE*, *tox1-tox2*, *relB-relE*, *higB-higA*, *graT-graA*, and *yefM-yoeB*; homologs of TA systems without proper names assigned include HTH-PIN, RHH-GNAT, *xre-relE*, *xre*-PIN, and *xre*-bro which were uncovered in one study (Andersen et al., 2017).

#### tomB-hha

The *tomB-hha* is a system that has been reported on *P. aeruginosa* (Fernández-García et al., 2016) with no reference sequence given. In this study, no homologs can be identified in the selected HTS data. Besides, even in the 5,423 genomes, only three gene hits were detected. Thus, we still characterized this TA module.

Two complete *tomB-hha* homologs were identified from two published draft genomes. The TA nucleotide sequences from both assemblies were highly similar to the corresponding reference NP_414993.1/NP_414994.1 from *E. coli* strain K12 MG1655 as the AIMHs reach 93.5/97.2% and 61.3/80.6%, respectively (Supplementary Table 8). Sequence alignment showed that a few amino acid substitutions were observed on the toxin Hha sequences but the conserved cysteine at position 18 (C18) is present (Supplementary Figure 5A). Also, the predicted protein structures of these homologs are similar to NP_414993.1 (Supplementary Figure 5B), which indicates that the amino acid substitutions do not make a huge difference to high dimensional structure formation, which is crucial to the interaction between YomB (TomB homolog from *Yersinia*) and Hha (Marimon et al., 2016). The C18 is an essential residue for the antitoxin–toxin interaction (Marimon et al., 2016), combined with the putative functioning protein structure mentioned above; there is a high possibility that the actual product of these Hha homologs carries similar toxicity as the Hha NP_414993.1.

The TomB sequence identified on contig JUPS01002551.1 (from GCA_001067615) is highly similar to the NP_414994.1. Two cysteine residues, C18 and C124 were observed in the TomB sequence of JUPS01002551.1. Though they conserve residues of the TomB family, these are not essential for the antitoxin activity of YomB toward Hha (Marimon et al., 2016). The four-helix regions of this homolog form a putative anti-parallel bundle (Supplementary Figure 5D), which is highly similar to the reference NP_414994.1. This hyperstructure is crucial for TomB-family acting as an antitoxin toward the Hha toxins. A similar structure was also observed on the TomB of CABFOI010001640.1 (from the assembly GCA_900683395) (Supplementary Figure 5D) though its liner sequence was much more similar to the other variant TomB reference, WP_004940312.1 (Marimon et al., 2016).

### Distinct pairwise kinship distance patterns among various toxin-antitoxin types

During the progression of this study, we noticed that a number of TA systems have not yet been reported in *P. aeruginosa*, e.g., the *hok-sok*. This finding drew our attention since some TA systems were reported to carry a narrow antibacterial activity spectrum (Fozo et al., 2010), though it is not always the case. We also noticed that the toxin component and the antitoxin component of the same TA systems were homologically different, as they matched references from various species. Hereafter, we introduced a statistics pKD to evaluate the relativeness between reference species and *P. aeruginosa* with the aims to (a) provide primitive hints to confirm the antibacterial spectrum of specific TA systems; (b) find out whether TA components were from different species, or, more conserved, to measure their homology.

With the assistance of pKDs calculated on all TA systems identified in this study, a rough correlation between the *P. aeruginosa* and reference species was observed: regardless of the type II TA systems, all references were of remote species. A dot plot was created to visualize the pKD of TA homologs; all pKD values of TA systems/orphans were either 8 or 12 (Supplementary Figure 7A), indicating that the LCAs were either at the kingdom level or the class level. These results were consistent with the findings uncovered by the literature survey that these TA systems were not likely reported on *P. aeruginosa*. Thus, this pKD method is likely to be capable of serving as a hint to confirm whether specific TA systems have been reported on query species or not before the paper survey.

The pKDs of type II TA systems present in a totally different pattern: the pKD of TA distributed from a wide range, ranging from 0 to 12; the pKDs of TA components were not always the same (Supplementary Figure 7B). This implies that the type II TA systems have a high degree of homology variation; the best match reference species could either be Pseudomonads or a remote species. The asynchrony of reference species of toxin component and antitoxin component of some identified TA systems indicate that these homologs (or the reference TA) may result from different phylogenetic sources, or, at least, they have different mutation rates.

### Characterization of toxin-antitoxin families in the *Pseudomonas aeruginosa* population

**FIGURE 3:**
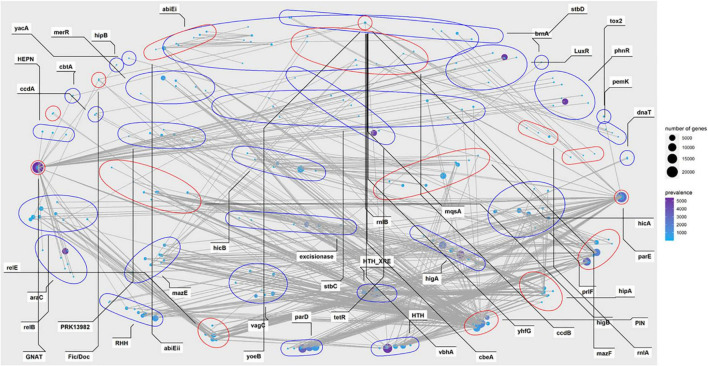
Prevalence network of the type II toxin-antitoxin (TA) grouped by gene family or domain in *P. aeruginosa* population. The nodes represent TA homologs from different loci, with locus names in circles. The size indicates the absolute count numbers of the corresponding locus. The prevalence of each locus (unique counts) among all strains are highlighted in the color scale. The red circles represent toxin components while the blue ones represent antitoxins. TA systems with a prevalence less than 10 were excluded.

**FIGURE 4:**
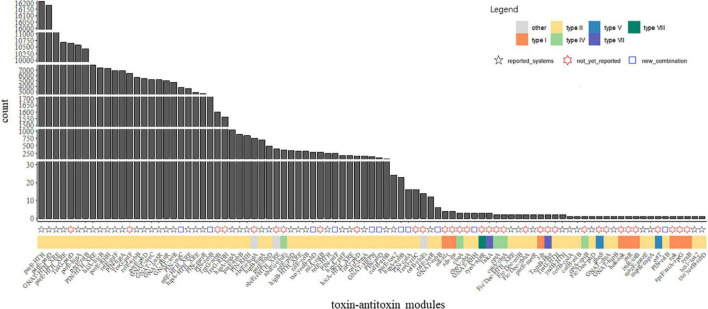
Abundance of the TA modules among *P. aeruginosa* population. Count indicates the absolute counts of TA homologs of specific systems, but with inner-strain deduplication. The names at the bottom were the identified TASs, the adjacent tiles were filled in colors which indicate the classification of the closest matched TA. The shapes indicate different labels of all TA systems: modules reported in the previous studies (Fernández-García et al., 2016; Andersen et al., 2017) were represented as black ✩; modules not yet reported in *P. aeruginosa* presents as red ✡; blue square indicates TA modules are new combination of TA with known domains.

Investigation of the combination revealed that huge different distribution of the TA families was observed in our data. Complicated combinations of toxin components and antitoxin components were observed in type II TA systems (Figures 3, 4). Most of the TA were already reported in the previous study, but there are a few new ones, as demonstrated in Supplementary Table 7 and Figure 4. This highlights the great diversity of type II TA systems and the flexibility of TA combination. Also, we uncovered some new TA domain combinations, highlighted in Figure 4, though the TA domain has already been demonstrated (Andersen et al., 2017). Besides the abundant type II systems, type I, IV, VII, and VIII were also uncovered among the 5,437 genomes/draft genomes.

### Annotation of mobile genetic elements and antibiotic resistance genes

**FIGURE 5:**
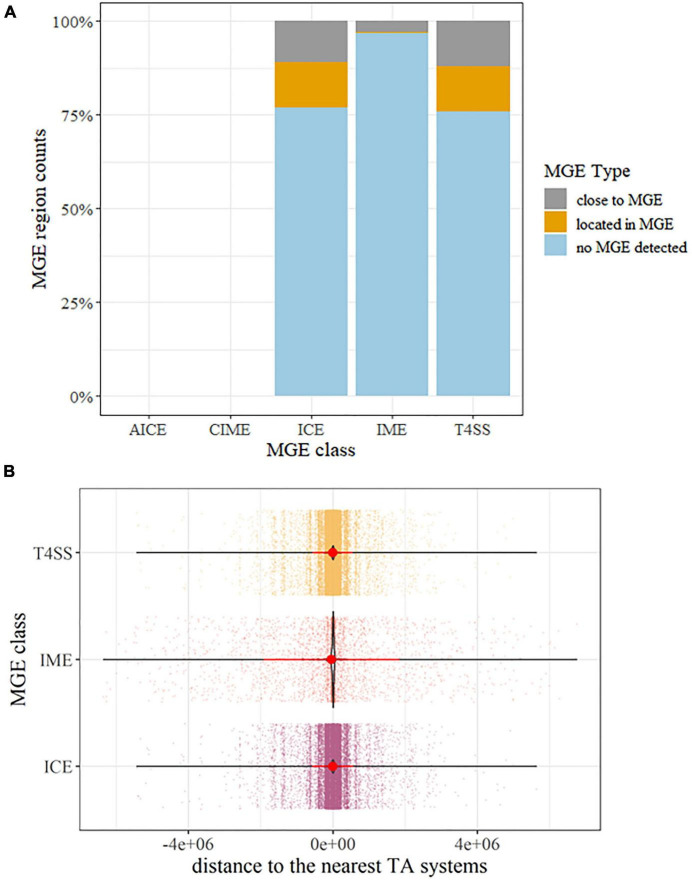
Overview of the identified mobile genetic elements (MGEs) associated with TA systems. **(A)** percentage barplot of MGEs detected on the same sequences carrying TA systems. Abbreviation: ICE, integrative and conjugative element; IMEs, integrative and mobilizable elements; AICE, actinomycete ICE, CIME, *cis*-mobilizable elements; T4SS, type IV secretion system type ICE. **(B)** Distance distribution of the MGE to the TA systems. Dots represent distances between TA and the nearest MGE on the same contig sequences or replicons. The violin plot highlights the condense distribution of MGE close to TA systems. Red dots are means and the line shows the range of standard deviation. If a TA was detected inside MGE regions, the distances were calculated as 0.

Mobile genetic element gene hits were identified in all *P. aeruginosa* strains. While no AICE and CIME homologs were detected, a large number of ICE, IME, and T4SS-type ICE homologs were detected, accounting for 23.03, 3.02, and 24.05% of the whole population (Figure 5A). Though a great number of sequences with TA hits had no MGEs detected (Figure 5A), all these sequences were short sequences under 500 bp; this may hinder confirming the relativeness between MGEs and putative TA. Thus, we further explore the physical distance between putative TA hits and detected the presence of MGE genes, 12.02%, and 12.19% of TA systems located within ICE and T4SS-type ICE, respectively. For the remaining putative TA homologs, most of them are located near the flanks of MGEs (−300,000 to +300,000 bp, while the MGE reference size range from 200 to 600,000 bp in ICEberg (Figure 5B). Combined with the fact that the genome size of *P. aeruginosa* is roughly 7,000,000 bp, this indicates that the putative TA gene hits were enriched on/near MGE hits instead of a random distribution over the whole genome. A similar situation could be observed in the ARGs. Of all the *P. aeruginosa* genomes, ARGs were detected in 5,416 strains; this covers almost all the samples included in this study. Similar to the MGEs, abundant detected ARGs are located close to the TA systems, as depicted in Supplementary Figure 8.

## Discussion

In this study, we conducted *in silico* TA identification on genomic public sequencing data, and then investigated the diversity in genome/draft genomes of a large population. Some methods were raised to automate the analysis of data on such a big scale.

The candidate new TA system, the dtas1_*hok-zok*, was uncovered in this study along with numerous divergent TA systems which have not yet been reported. These findings conclude our hypothesis that unexplored TA resources are in the pan-genome of *P. aeruginosa*.

The searching for TA uncovered plentiful TA systems in this study. These TA systems covered not only abundant TA systems highly similar to known systems but also some divergent or new TA. Type II were the most abundant TA systems unveiled in this study, accounting for 99% of the total matches in the first-round homology search; most of these type II TA systems homologs match the families revealed from a study that systematically explored the type II TA systems in *P. aeruginosa* in a small scale (Fernández-García et al., 2016; Andersen et al., 2017). However, to the best of our knowledge, the findings of type I and type IV TA systems identified in this study were likely not yet reported on *P. aeruginosa*.

The type I TA system, dtas1_*hok-sok-mok*, was unveiled by combining homology-based scan and rule-based inspection. The first-round homology-based scanning did not confirm any divergent form of *sok* or other antitoxins on the flank of dtas1_hok, but manual inspection of the exact physical distance to the start codon referenced to reported *hok-sok-mok* locus uncovered putative *sok* homolog. This manual inspection process was a comprehensive step combining ORF prediction, local sequence alignment, and secondary structure prediction through which an extensive literature survey was involved. The progression investigating these TA systems reflects the complicatedness of identifying RNA-protein TA systems, and it could be used to explain why the reports and reference sequences of type I TA systems are much less than those of type II TA systems, though it might be a small part. It is a pity that there are no robust tools for type I or type III *de novo* prediction and only reference-based tools were available (Sevin and Barloy-Hubler, 2007; Akarsu et al., 2019). It is unclear whether the product of dtas1_hok functions as a proper toxin or not. Though it carries the conserved cysteine 31 which is required for retaining toxicity (Supplementary Figure 2B), the SWISS-model prediction showed that the periplasmic C-terminal of dtas1_Hok helix is shorter than a normal Hok (Supplementary Figure 2C), which is important for Hok’s activity (Wilmaerts et al., 2021). No precise complementary regions of *sok* RNA and *hok* mRNA could be found, which increases the uncertainty of the *sok*-like being as a qualified antitoxin.

The dtas4_*ralD-ldrD* is another type I TA homolog identified in this study. It is reported that *ralD-ldrD* presents in 4 copies in the genome of *E. coli* K12; among which three are in large direct repeats (LDRs) (the LDR-A, LDR-B, and LDR-C), and one is in a single copy (LDR-D) (Kawano et al., 2002). The *ldrD* mRNA sequence of dtas4 is present only in a single copy, and no other LDR is found on other contigs. While the dtas4_*ralD* shows relatively high identity to the corresponding reference *ralD* (TADB| AT6339) found in *E. coli* K12), the toxin dtas_ldrD does not. Considering the toxin sequence only shared a low identity with known references, we further inspect the possible impacts of the SNPs on its function.

It should be mentioned that all type I toxins are hydrophobic peptides sharing a signature α-helix *trans*-membrane domain (Nonin-Lecomte et al., 2021). The hydrophobicity pattern and the predicted α-helix (Supplementary Figures 4C–E) suggested that dtas4_LdrD is highly similar to LdrD TADB| T6339, which has been experimentally validated (Kawano et al., 2002); this indicates that dtas4_LdrD may locate on the membrane with high affinity. Further, the identical starting amino acid on the N-terminal to reference TADB| T6339 is another hint supporting that the dtas4_LdrD may be toxic since the extracellular N-termini is essential (Yamaguchi et al., 2014). However, the residue difference, especially on the C-terminal may negatively influence the activity of dtas4_LdrD. It is reported that the LdrD C-terminal is predicted to locate in the cytosol, which depends heavily on the positively charged residues on C-terminal (Scheglmann et al., 2002). The residues on the C-terminal of dtas4_LdrD are “WLNKRK,” whose charges could be sequentially present as “000+++” (0 indicates no charge), while the C-terminal residues of dtas4_LdrD are “+0-0++.” It is pointed out that the toxicity is dependent on the ending cationic amino acids on AapA1 (Korkut et al., 2020); this may also apply to LdrD since the last two ending amino acids are conserved. The introduction of the negatively charged “D” on position 32 may diminish the overall charges of the dtas4_LdrD C-terminal, and further, it diminishes the binding ability of dtas4_LdrD with lipid bilayers on the membrane. Besides, the effect of two variances of conserved residues on positions 12 and 30 and the many residue substitutions on non-conserved sites (Supplementary Figure 4B) was unclear. Finally, start codons were not found in the downstream adjacency (300 nt) of dtas4_ralD (the promoter *ldrD* mRNA in *E. coli* locates at ∼190 nt of *ralD*); also there was no sign of a region similar to the promoters reported in *ralD-ldrD* loci (Kawano et al., 2002). A couple of TSSs were confirmed in this region using a promoter prediction function designed for *Pseudomonas* in SAPHIRRE2 (Coppens and Lavigne, 2020) and we kept three as putative promoters for *ldrD* (Supplementary Figure 4A). Whether the dtas4_*ralD-ldrD* could be properly transcribed, the possible effect of the residue substitutions on LdrD’s toxicity, and the lethality of toxin product against *P. aeruginosa* were interesting questions that deserve further investigation in future studies.

The IV TA systems of *cbeA-cbeB* homologs, the dtas351_*cbeA-cbeB*, were identified in this study. Our analysis identified some SNPs in the toxin genomic sequences of the dtas351, but secondary structure prediction implies that they do not prohibit the N-terminal of amino acid from forming a functional helix structure. However, numerous amino acid replacements were also observed in the C-terminal and their impact on the toxin remains unclear. The transmembrane regions of CptA formed a helix structure that plays a key role in the CptA subcellular localization, but the toxicity seemed to be determined by the C-terminal since the N-terminal helix structure binds the lipidic membrane leaving free C-terminal stretching in the cytoplasm (Masuda et al., 2012). The *cptA-cptB* was reported for the first time in 2012 (Masuda et al., 2012), but only a few studies were associated with these loci, and how the C-terminal acts in the mechanism are unclear. Besides, though abundant homologs were identified in *E. coli*, *Acinetobacter baumannii*, and some other species (ElBanna et al., 2021), these loci have not yet been reported in *P. aeruginosa*. The actual role of *cptA-cptB* in *P. aeruginosa* requires future study to provide hints to interpret.

Besides the TA modules mentioned above, we also identified several homologs belonging to type VII and type VIII TA, which are two systems established in recent years. These homologs have high similarity to the reference sequences and solid genetic evidence to prove they are qualified TA systems. Based on the analysis of the conserve/essential residues and protein structure, it could be assumed that the protein product or sRNA product of the TomB, Hha, and sdsR homologs may be functional. However, a question was raised about the type VII and type VIII homologs regarding their location in the actual species, *P. aeruginosa*. The two complete homologs *tomB-hha* and *ryeA-sdsR* homologs were detected on the same two assemblies, the GCA_001067615, and the GCA_900683395, though they are found on different sequences. By investigating the metadata, the biological samples of these assemblies were confirmed to be *P. aeruginosa* isolates from human samples. However, they both present a big genome size, 12.3 and 11.9 Mb, respectively, which are an abnormal size for *P. aeruginosa* (roughly 7 Mb). We also noted that another assembly GCA_001181985.1 has a genome size of 11.85 Mb although no qualified type VII/VIII gene hits were detected. Interestingly, these three assemblies have roughly the same genome size but from distinct geographical sources, (England, United States, and Spain), and it is not like they are just simply contaminated. Though results from MetaPhlAn3 confirmed that the contigs carrying these TA modules were classified as *P. aeruginosa*, it cannot be concluded that these type VII and type VIII homologs were detected on *P. aeruginosa*, or another unknown species close to *Pseudomonas*.

The distinct pKD pattern results of type II and the TA systems of other types is an interesting finding. Technically, this may be a result introduced by using an unbalanced reference database since most of the records of TADB2 were type II TA systems. The type II subset of TADB2 contained a large number of homologs uncovered by *in silico* homology scan and iteration (Xie et al., 2018); thus a great diversity is expected in the type II references which may lead to biased results. To overcome this, hits from *P. aeruginosa* were prime selection during the step of the best match calling, but asynchrony was still observed. To conclude a distinct introduction of any asynchronous TA systems, additional evidence is required. Nevertheless, this method provides a stat to overview the relativeness of the reference species against the query species, which provides primitive hints to link the undocumented TA systems to the species of interest and increases the efficiency of large-scale data mining.

The pKD method has promising prospects in the other aspects. The presence of a toxin homolog matching reference from remote species, if available, does not just expand our knowledge of this toxin. It is also a marker of the antibacterial activity of the host species which indicates a shared mechanism between them. The presence of mechanism usually indicates common traits; these include but are not limited to substrate, intermediate, structure, and motif. Further investigation into this information, combined with the knowledge of the reported mechanism, may lead to new findings, such new rule-based method to predict pathways on new query species and further, confirm the antibacterial activity. Another scenario is deploying the pKD in metadata analysis, such as exploring the diversity of wild-type strains, and the HGT acquisition of TA systems from a metagenome in different niches.

In a previous study, great diversity and prevalence of type II modules, grouped by families or domains, were observed (Andersen et al., 2017). While our results roughly recovered the prevalence pattern of this study in *P. aeruginosa*, some new combinations of reported gene locus were observed, including but not limited to GNAT-RHH, GNAT-arbB, *hipA*-arbB, PIN-relB, and *txe/yoeB-phD* (Figure 4). Regarding TA types, type II TA was the most abundant class in *P. aeruginosa*, while other types of TA were scarcely observed in the population. The components *parD*, *parE*, *relB*, *relE*, GNAT, HTH_XRE, PIN *higB*, and *higA* were the most abundant among all type II gene loci, as their count is not only the most of all after sorting (Figure 4), but also their connection with other components was the most intensive (Figure 3).

A group of TA modules, with all the toxin components, are TraG, were also present in great numbers in the 5,437 genome or draft genomes. The top 4 abundant Trag-X TA modules are TraG-*parD*, TraG-*prlF* (*prlF* is a homolog of *mazE*), TraG-RHH, and TraG-*arbB* which are 1,0651, 6,405, 1,565, and 1,004 count in total, individually. All TraG hits refer to a group of reference sequences which are all *in silico* predicted toxins recorded in the TADB2 database. Thus it is uncertain whether their products are toxic. Another interesting finding is a group of *abiEii*-X modules. This group includes the *abiEii-abiEi* homolog with a total count of 358 in *P. aeruginosa* population, which is a well-established type IV TA system (Kuchta et al., 2009); thus these groups present as a typical type IV system. However, the *abiEii* plus another type II domain/gene were observed, including *abiEii*-HTH_XRE (401 counts in total) and *abiEii****-****higA* (771). Also, two type IV-antitoxin with type II toxin combination, the *relE-abiEi*, and GNAT-*cbtA* were observed, but their count were as small as 14 and 1, individually. It is unsure how to categorize these modules, such as the combo of TA was labeled as “others” in Figure 3. We inspected the nearest hit references of these type II–type IV combos and found that they were all computationally predicted records in the TADB database but assigned as in the type II TA systems. It was during the CDD scanning step in our workflow that the type IV label was assigned. These results showed that they were closer to type IV TA systems, but whether they are actual mis-annotation or crossover needs further evaluation.

The few non-type II systems uncovered in the 5,437 genomes were also interesting findings. Besides the *abiEii*-X modules as mentioned above, the type IV homologs, *cbeA-cbtA*, *cptA-cbtA*, and four putative orphans were uncovered in the 5,000 = strain population. But they are limited in several strains (GCA_001067615, GCA_001180405, GCA_900683395) and all these strains were draft genomes (contig level) (Supplementary Table 8). Though the result of MetaPhlAn3 indicates they are of *P. aeruginosa*, the result from blastn showed that the sequences carrying these TA modules do not match with any *P. aeruginosa* genomes (complete). A similar situation could be observed in other systems, including the type I, type VII, and VIII modules. We manually inspected all sequences and found that all candidate orphans were located on small contigs (≤1000 bp). Considering their small number in population and uncertainty to conclude their source (either bacteriophage/plasmid or chromosome, including the free forms and integrative elements), the identity of these homologs requires further evaluation.

In this study, we uncovered ample genomic sequences not only limited to chromosomal sequences but also those belonging to putative plasmids and putative viruses. This is due to the advantage of the HTS, from which the generated data has the feasible ability to contain all sorts of genomic elements. But the power of HTS on data mining has not been fully unleashed. The isoforms of genes of interest were not explored in this study and it is usually overlooked in other studies, but it is proved to be capable to uncover isoforms and used in diversity research (Dai et al., 2019). Another key advantage of HTS data is that they are reusable; new knowledge was expected to uncover through data reanalyzing as the reference database size keeps on increasing. Given the fact that the TA reference database is heavily unbalanced (Xie et al., 2018), the reported RNA-protein TA systems are much less than the protein-protein TA systems and they are reported on a limited group of bacteria, it is expected that new advances could lead to expansion of this TA family.

It is noted that other types of genetic elements involving antibacterial activity have not been explored; these include but are not limited to the genes coding lytic enzyme/proteins, anti-biofilm compounds, and abortive infection system. Considering the discovery of abundant TA systems in this study, we assume that abundant homologs or new systems belonging to these gene types could be found. Besides, more than 100 strains were involved in this study. The findings were based on data on a limited scale, an exhaustive exploration of all available *P. aeruginosa* data is difficult to achieve, let alone the increasing size of available sequencing data, and the reference sequences uncovered by advanced research. It is expected that novel findings could be found in future studies based on data on a larger scale.

A large number of TA and ARG are located in or close to MGEs, as depicted in Figure 5 and Supplementary Figure 5. It should be also noted that though a high percentage of total genome sequences that have no MGE were observed (Figure 5A), 86% of these were indeed short sequences (less than 3000 bp) belonging to draft genomes (contig or scaffolds level). The low assembly level may hinder mining the connections of MGE/ARG and TA on the same sequences and lead to a biased result. This also applies to ARG, though almost all 5,437 strains carry ARG, only 9.84% of all ARG homologs were detected along with TA and MGE at a close distance (Supplementary Figure 5). Thus, it cannot be excluded that improving the assembly of these strains may also have MGE/ARGs associated with TA. The connection between MGE and ARG has been reported (Stokes and Gillings, 2011); the TA forms triple interaction with them and makes these connections more complicated. The MGE is a key driving force mediating the dissemination of TA and ARG (Stokes and Gillings, 2011; Koonin et al., 2020). The TA was reported to stabilize MGE on the chromosome (Wozniak and Waldor, 2009), and it is also reported to maintain AR; this indicates that TA could assist in disseminating ARG in an indirect way (Yang and Walsh, 2017). The presence of ARG helps the small population of bacteria with newly introduced MGE or TA survive and eventually become dominant in the whole population in response to antibiotic stress. Thus, the TA, MGE, and ARG play individual roles in this triangle connection. Any strains with all these three elements may increase the dissemination rate of ARG, which is a crucial trait for AR development for clinical pathogen bacteria. In a recent large-scale genome mining study, a group of MGE with TA and ARG detected gut pathogens and symbionts in which mobiles cross taxa at the phylum level (Forster et al., 2022). A summary table of all ARG annotations is provided in Supplementary Table 9.

## Conclusion

In this study, we uncovered a great number of TA systems through *in silico* identification of genomic public sequencing data of *P. aeruginosa*. A wealth of divergent TA homologs was identified on the chromosomal, putative bacteriophage/plasmid regions, and other MGEs. The new type II TA combinations were an extension of our knowledge of the type II systems in *P. aeruginosa*, while most of the non-type II TA systems have not yet been reported in *P. aeruginosa*. Besides, a large number of TA homologs are located close to the MGEs and ARGs on the genomes, which indicates an interaction among these three genomic elements. These new findings expanded our knowledge of *P. aeruginosa* and conclude the great prospect of TA mining from the unexplored bacteria pan-genome. Our methods provide prime knowledge and experience in TA findings based on large-scale data mining. The additional experimental proof is required to confirm the production capability of the identified TA systems and the bioactivity of the corresponding product.

## Data availability statement

The original contributions presented in this study are included in the article/Supplementary material, further inquiries can be directed to the corresponding author.

## Author contributions

ZD was the major contributor who made substantial contributions to the conception, data collection, and manuscript writing of this project. TW, SX, LaZ, and WT provided the crucial technical support to this study. EH, LiZ, and MC provide support in data visualization for this study. GY as the supervisor of this study, provide support in all aspects throughout the progression of this study, and approved the final version of the manuscript. All authors contributed to the article and approved the submitted version.

## Footnotes

1
https://github.com/IBM/aspera-cli

2
https://swissmodel.expasy.org/

3
https://blast.ncbi.nlm.nih.gov/Blast.cgi

4
https://pypi.org/project/taxonomy/
