# Supplementary material for: Characterization of toxin-antitoxin systems from public sequencing data: A case study in Pseudomonas aeruginosa
Source: Front Microbiol. 2022 Aug 16;13:951774. doi: 10.3389/fmicb.2022.951774 (PMC9424990; doi:10.3389/fmicb.2022.951774)
Supplement: Supplementary file 1 [file Data_Sheet_1.docx]

Supplemental Figures of

**Characterization of toxin-antitoxin systems from public sequencing data: a case study in *Pseudomonas aeruginosa***

**Zehan Dai^1^, Tianzhi Wu^1^, Shuangbin Xu^1^, Lang Zhou^1^, Wenli Tang^1^, Erqian Hu^1^, Li Zhan^1^, Meijun Chen^1^, Guangchuang Yu^1,^***


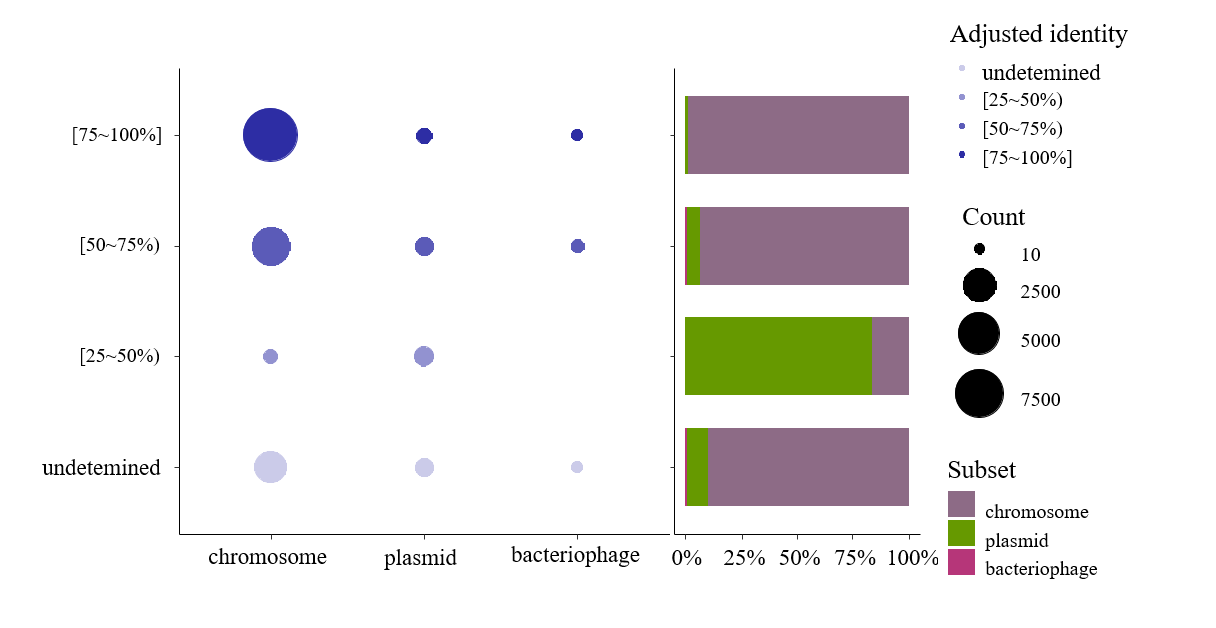


Figure S1. Distribution pattern of identified TA systems grouped by category and similarity.


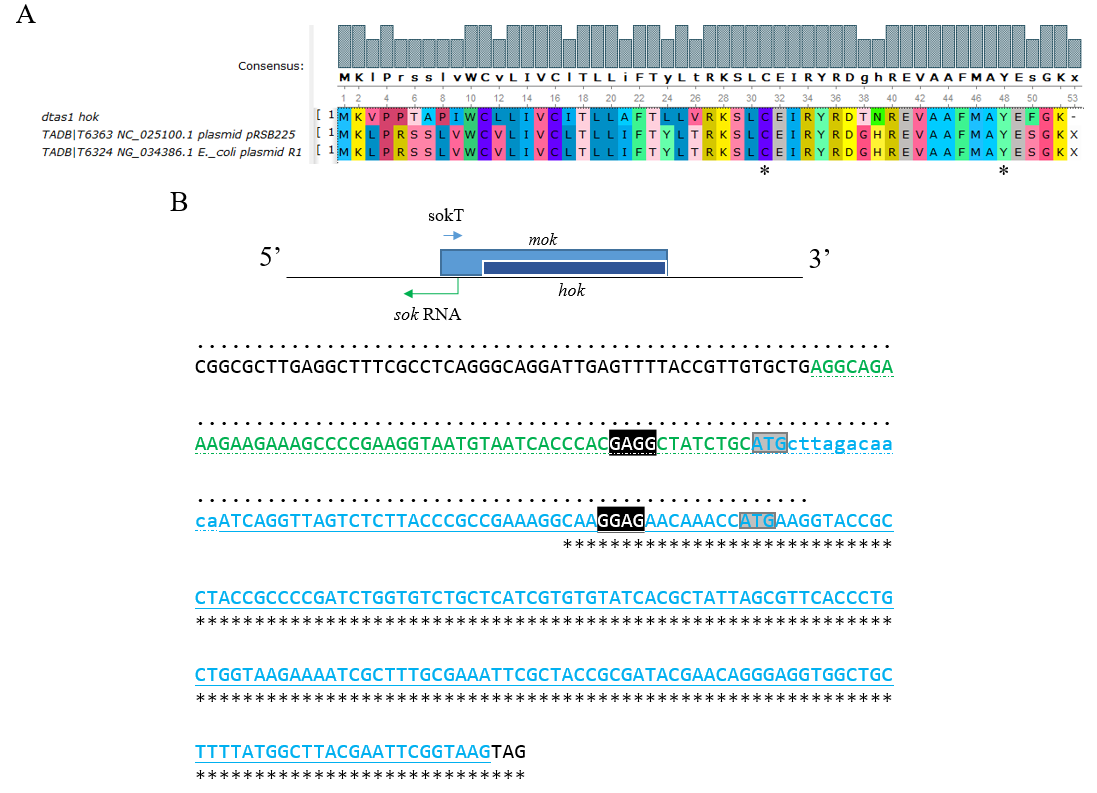


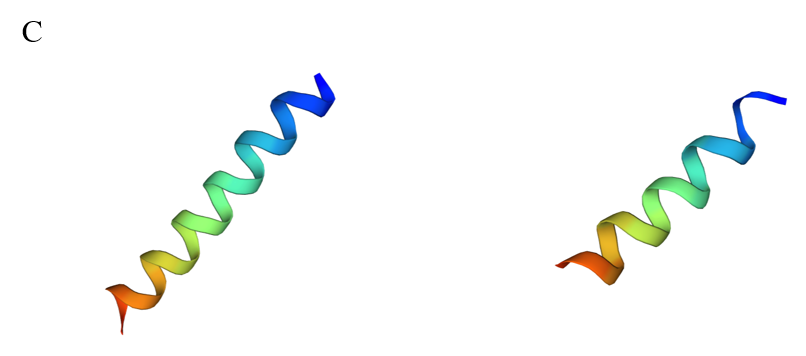


Figure S2. Additional information of *hok-sok-sok* locus. A. Sequence alignment of the amino acid sequences of Hok. The TADB|T6363 and TADB|T6324 were Hok sequences recorded in TADB2 database. The asterisk marks indicates the conserve residues. B. Schematic presentation and sequence of dtas1_*hok-mok-sok* locus. Region of the *hok* homologue was showed with asterisks (*); region of the *sok* homolog was shown with dot (.), the putative *sok* RNA region was underlined with dot lines, regions present in lower letter was the *sok* target region (sokT); sequence highlighted in blue was the *mok* homolog which contains most of the *hok* and overlap with *sok* homolog. The start codons were shaded in grey while the Shine-Dalgarno regions on the TIR regions was shaded in black. C. Predicted protein structure of *Hok* protein*.* The left one is TADB|T6324, the right one is dtas1_Hok.


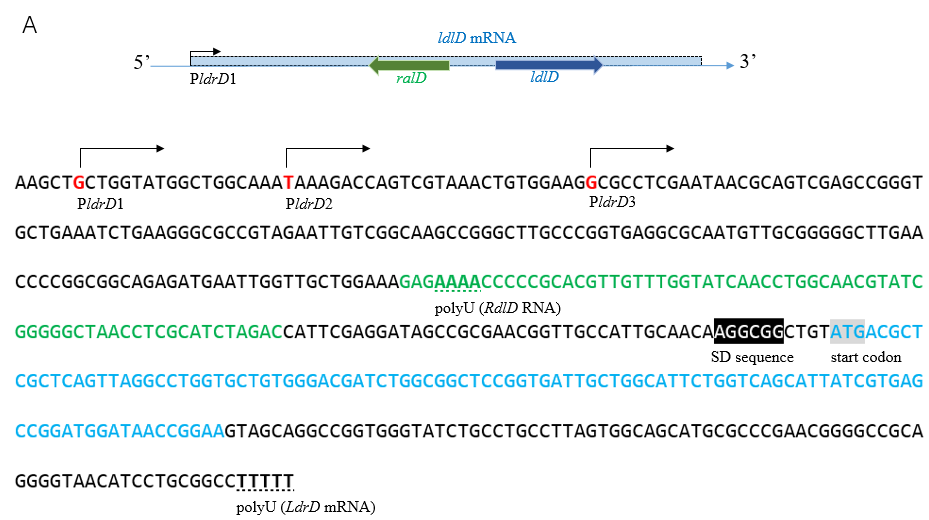


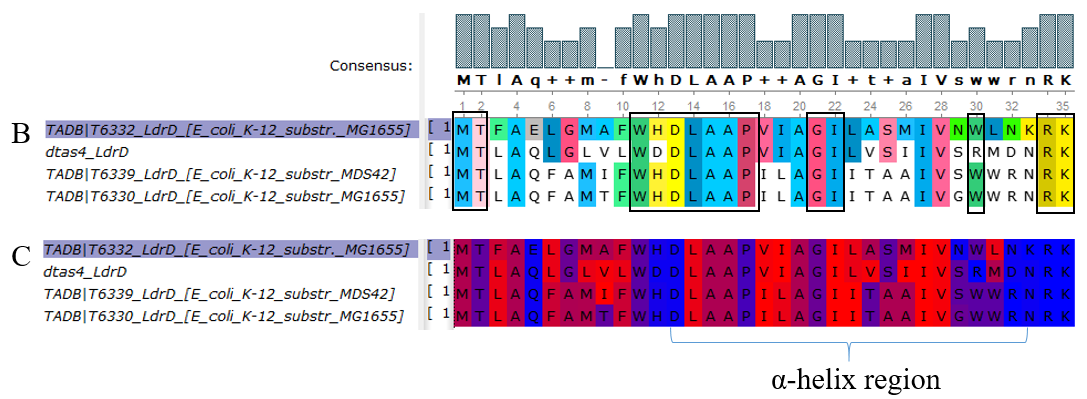


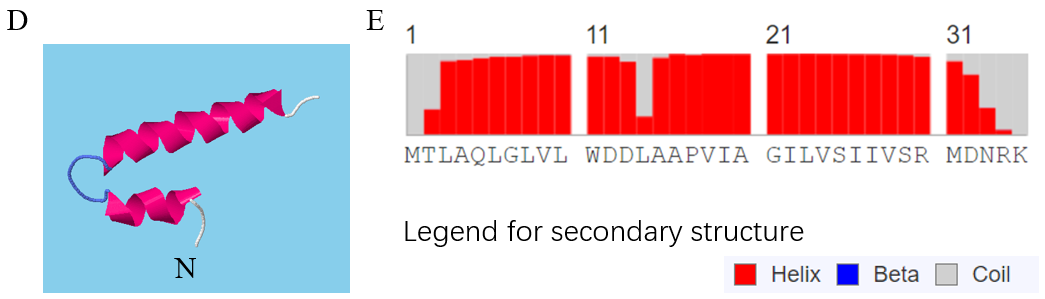


Figure S3 Additional information of *ralD-ldrD*.

A. Schematic presentation and genomic sequence of dtas4_*ralD-ldrD* locus. P*ldrD*1 is the putative promoter of *LdlD* mRNA, as the first letters highlighted in red, the PldrD2 and P*ldrD*3 are alternative promoters. The promoters were predicted using the “promter predictor for *Pseudomonas*” function implemented in SAPPHIRE 2 (Coppends et al., 2020) with P-value cutoff as 0.01. The *ral*D gene sequence is highlighted in green while the *ldrD* gene is highlighted in blue. The putative SD sequence is shaded in black and the start codon of *ldrD* gene in grey. The putative poly U signal of LdrD mRNA and RdlD antisense RNA are underlined with dashed lines. B. Sequence alignment of the amino acid sequences of LdrD gene. The TADB|T6332, TADB|T6339, and TADB|T6330 were reference LdrD sequences recorded in TADB2 database while the dtas4_LdrD was a homolog identified in this study. Sequence alignment was visualized in UGENE using color theme highlighting conservation level. The TADB|T6332 was set as reference. The conserved amino acids documented in previous study (Kawano et al., 2002) was boxed. C. Sequence alignment of the amino acid sequences of *LdrD* gene using color theme highlighting in hydrophobicity. The braced region refers to the transmembrane regions of reference with α-helical structures which was validated by experiment (Göbl et al., 2002). D and E, 3d model and sequential view of predicted secondary structures of dtas4_LdrD generated by RaptorX (Peng et al., 2011).


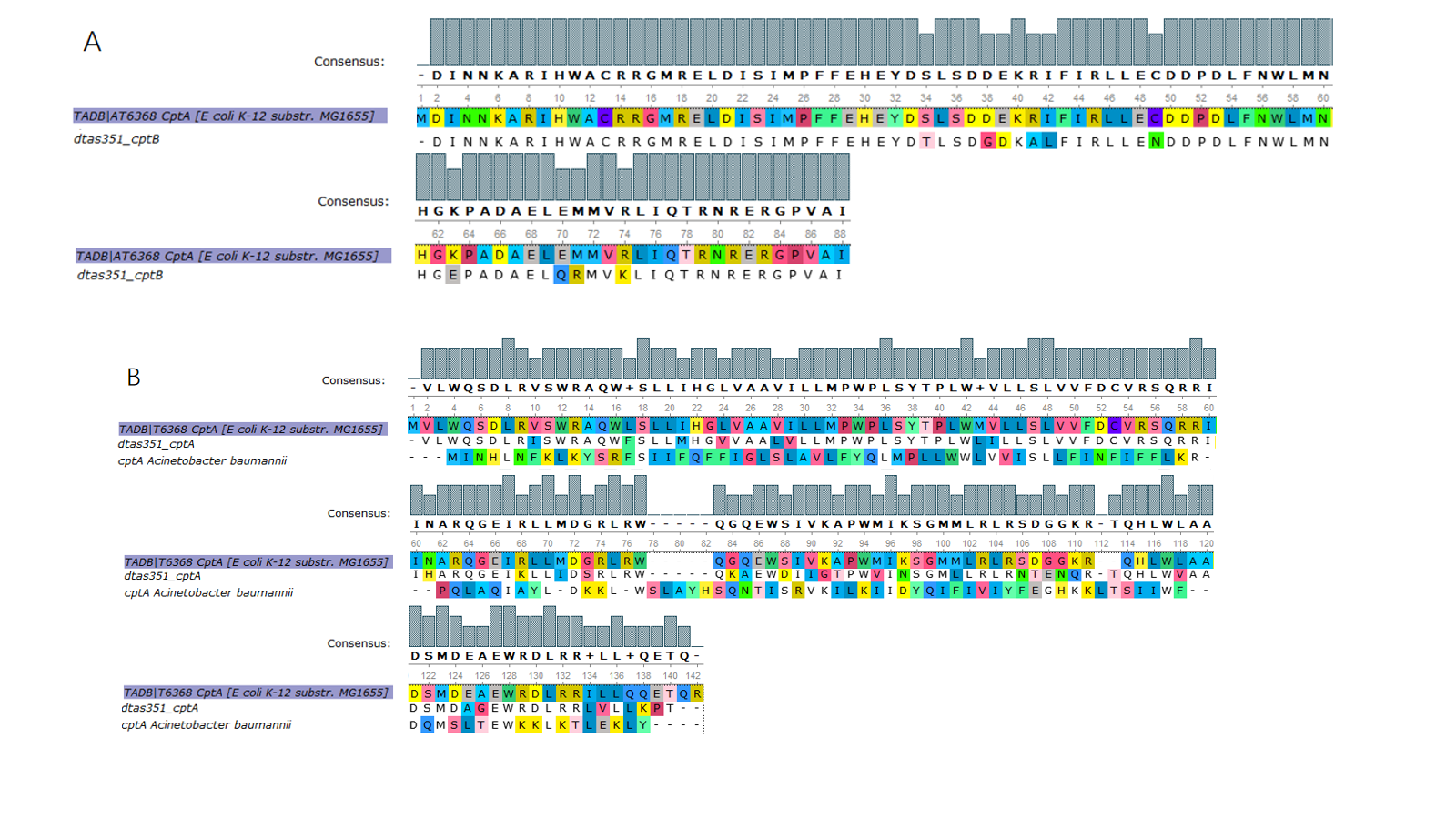

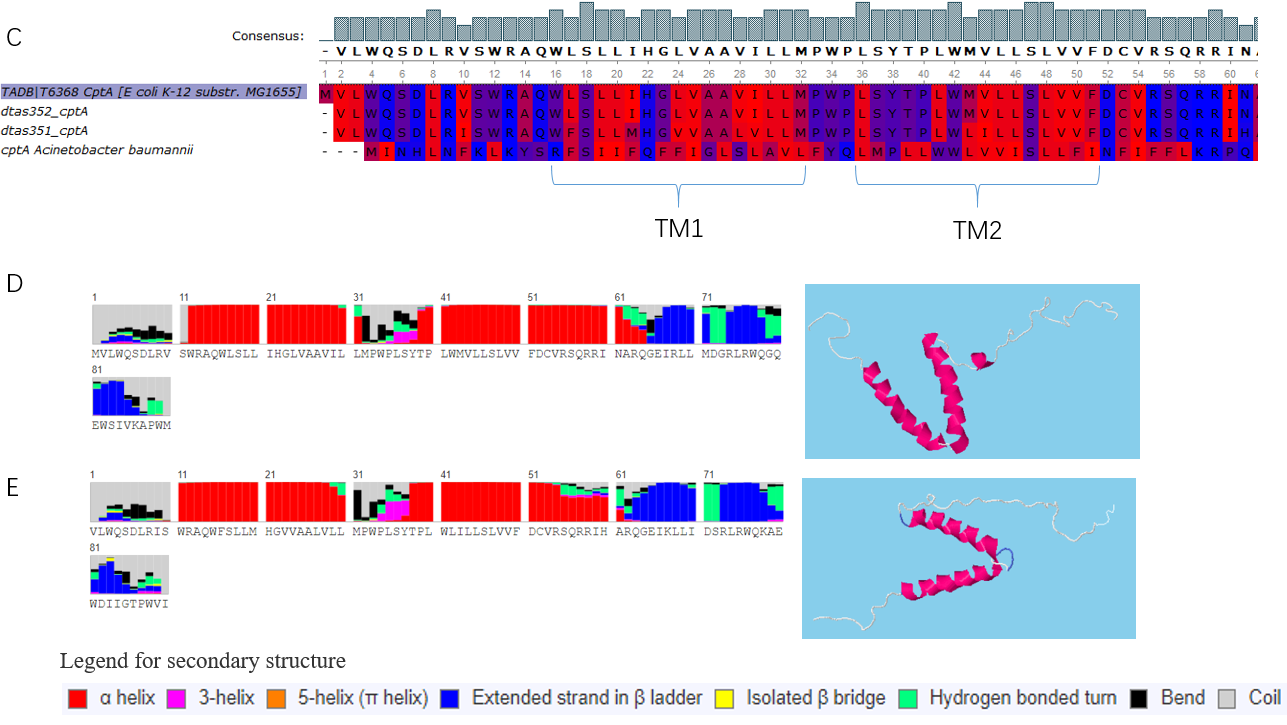


**Figure S4. A**. Sequence alignment of CptB. **B**. Sequence alignment of antitoxin CptA. The amino acid sequence of CptA (TADB|T6368) and CptB (TADB|AT6368) were set as refererences, SNPs were highlighted based on the amino acid. The position, consensus sequence and consensus score were plotted on the top of sequence area. The *cptA* homolog of *Acinectobacter baumannii* ATCC 17978 was curated from a recent study (ElBanna et al., 2021). **C**, Alignment of CptA toxin homologs with hydrophobicity color theme. Only the 1~60 aa region was included, showing the trans-membrane region. The nucleotides were highlighted based on the hydrophobicity of each amino acid where color scale from blue to red indicates the hydrophobicity range from weak to strong. The Bros at position 33 and 35 and the neighboring region with hydrophobicity indicated a typical transmembrane structure. TM1: transmembrane domain 1; TM2, transmembrane domain 2. **D** and **E**. The predicted secondary structure and 3D models of cptA toxin homologs dtas351_CptA.


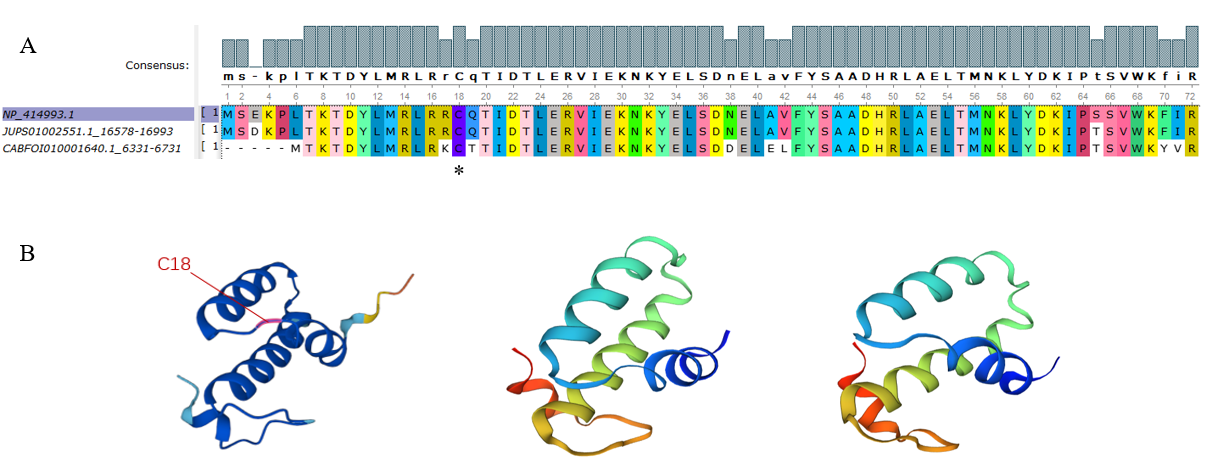


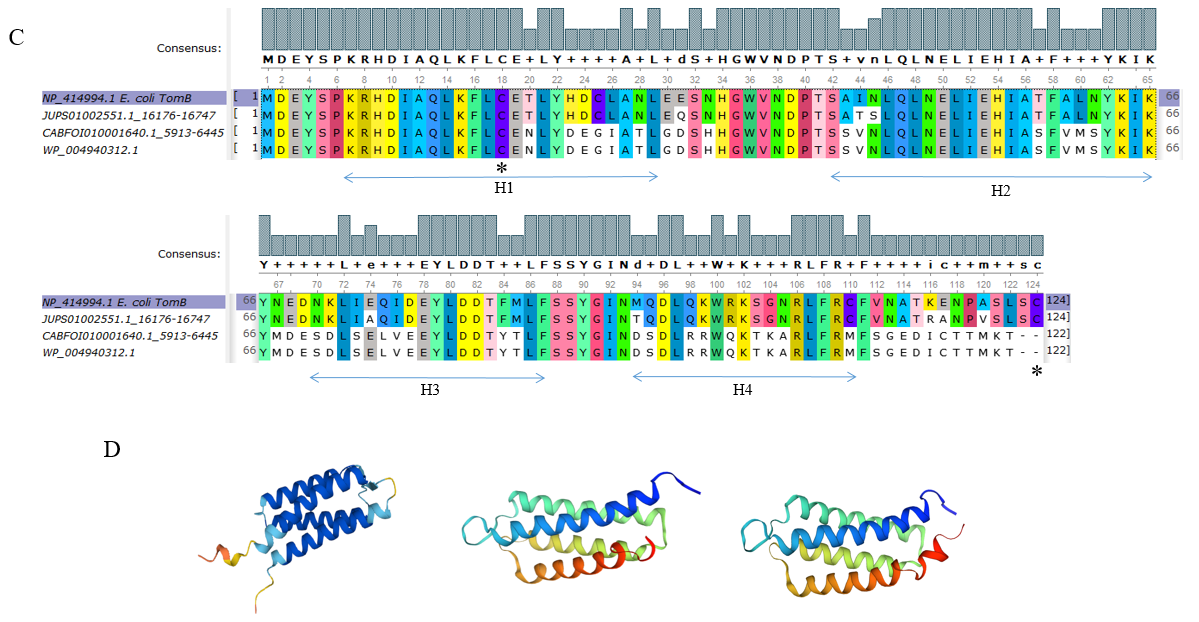


**Figure S5 Additional information of *tomB-hha* homologs**.

**A**. Sequence alignment of Hha amino acid sequences. The NP_414993.1 is the reference protein sequence from E. coli strain K12 while the others are homologs identified in this study. The sequence JUPS01002551.1 is from the assembly GCA_001067615 and the sequence CABFOI010001640.1 is from the assembly GCA_900683395. The asterisk marks indicates the conserve residue. **B**. The three predicted protein structures of Hha from left to right are NP_414993.1, JUPS01002551.1_16176-16747, CABFOI010001640.1_5913-6445, respectively. The structure of NP_414993.1 was retrieved from Uniprot. The coloring in the NP_414993.1 is computed confidence score in Uniprot and carrying no meaning in this study. Predictions of other homologs were conducted with SWISS-MODEL, the rainbow color theme only represents the sequences order, starting from 5’ to 3’. **C**. Sequence alignment of the translated TomB amino acid sequences. The conserve cysteine residues were marked with asterisks. The TomB sequence from *E. coli* (NP_414994.1) was selected as reference. The WP_004940313.1 is variant TomB homolog curated from a previous study (Marimon et al., 2016). Four helix regions in TomB gene **D** The three predicted protein structures of TomB from left to right are NP_414993.1, JUPS01002551.1_16176-16747, CABFOI010001640.1_5913-6445, respectively.


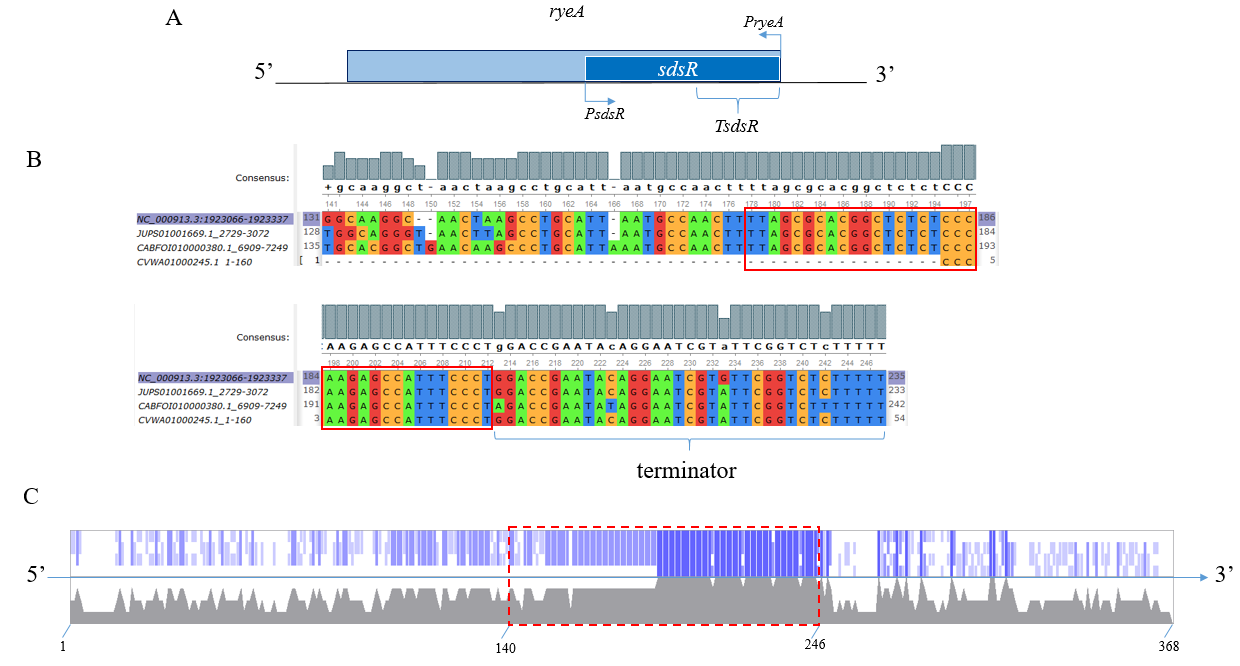


**Figure S6 Additional information of of the *ryeA*-*sdsR* homologs.**

A. Schematic presentation of the genetic structure of *ryeA-sdsR* module. The PsdsR and PryeA indicates the startings of the *sdsR* and *ryeA*, respectively. The TsdsR indicates the terminator of the *sdsR* locus.

B. Nucleotide sequence alignment of the toxin *sdsR*. The essential region for retaining the toxicity is highlighted in red box while the other regions is dispensible (Choi et al., 2018). The terminator region is bracketed. The sequence NC_000913 is selected as reference.

C. Overview of sequence alignment of the whole ryeA loci. The upper graph is the identity heatmap where the consensus nucleotide is highlighted in blue color. Positions with deeper blue indicates these positions have higher average identity value, nucleotides differ from the consensus is colored in white. The below graph is the conservation plot where the gray area indicates the conservation level on each column .


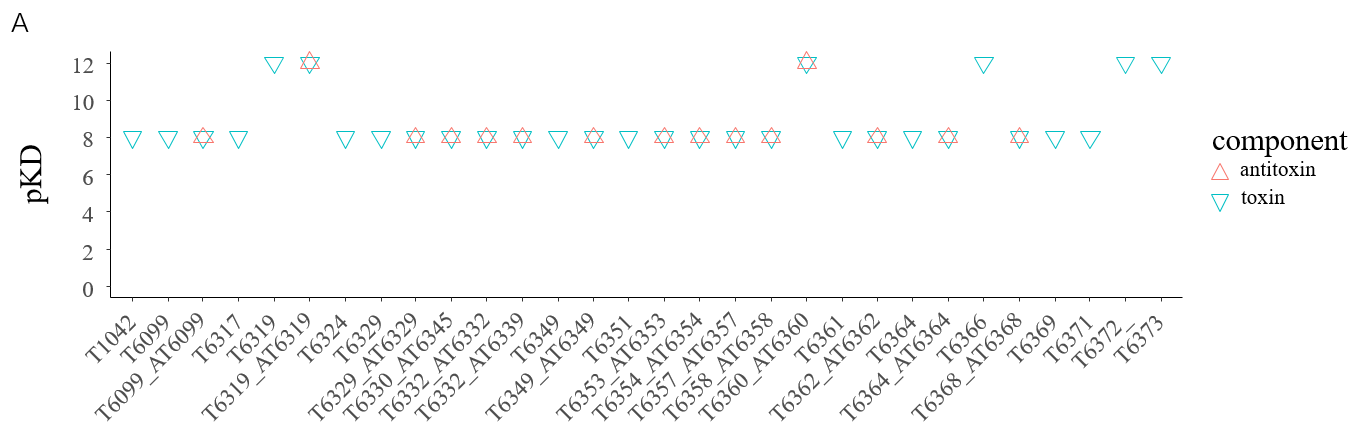

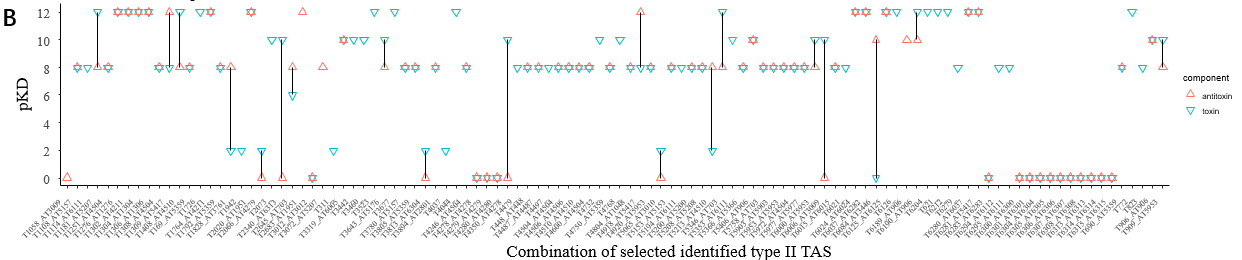


Figure S7. Relativeness of matched reference toxin/antitoxin against *Pseudomonas aerginosa*. A. pKDs calculated on all reference TAS with experimentally proofs. B pKDs calculated based on selected 100 identified type II TASs.


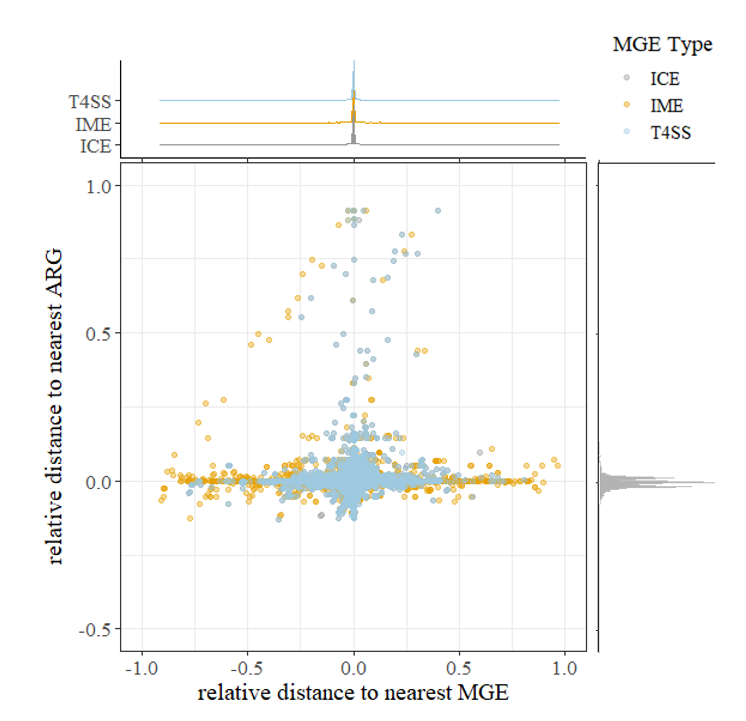


Figure S8 Distance of the MGE and ARG to the TA homolog. The dots represent distances from TA to nearest MGEs or ARGs, which are normalized by dividing each genome sizes. The top and right density ridge graphs shows the location preferences of the detected MGE and ARG homologs against TA systems, respectively.
